# Advancing a conceptual model to improve maternal health quality: The Person-Centered Care Framework for Reproductive Health Equity

**DOI:** 10.12688/gatesopenres.12756.1

**Published:** 2017-11-06

**Authors:** May Sudhinaraset, Patience Afulani, Nadia Diamond-Smith, Sanghita Bhattacharyya, France Donnay, Dominic Montagu

**Affiliations:** 1 Global Health Sciences, University of California, San Francisco, San Francisco, CA, 94105, USA; 2 Community Health Sciences, Fielding School of Public Health, University of California, Los Angeles, Los Angeles, CA, 90095, USA; 3 Public Health Foundation of India, Delhi, 122002, India; 4 Tulane University School of Public Health and Tropical Medicine, New Orleans, LA, 70112, USA

**Keywords:** Maternal Health, Reproductive Health, Quality, Equity, Health Seeking Behavior, Quality of Care, Person-Centered Care, Patient Experience

## Abstract

**Background:** Globally, substantial health inequities exist with regard to maternal, newborn and reproductive health. Lack of access to good quality care—across its many dimensions—is a key factor driving these inequities. Significant global efforts have been made towards improving the quality of care within facilities for maternal and reproductive health. However, one critically overlooked aspect of quality improvement activities is person-centered care.

**Main body:** The objective of this paper is to review existing literature and theories related to person-centered reproductive health care to develop a framework for improving the quality of reproductive health, particularly in low and middle-income countries. This paper proposes the Person-Centered Care Framework for Reproductive Health Equity, which describes three levels of interdependent contexts for women’s reproductive health: societal and community determinants of health equity, women’s health-seeking behaviors, and the quality of care within the walls of the facility. It lays out eight domains of person-centered care for maternal and reproductive health.

**Conclusions:** Person-centered care has been shown to improve outcomes; yet, there is no consensus on definitions and measures in the area of women’s reproductive health care. The proposed Framework reviews essential aspects of person-centered reproductive health care.

## Introduction

Every day, 830 women die from preventable causes related to pregnancy and childbirth, with 99% of all deaths occurring in low and middle-income countries (LMICs) ^1^. Poor quality care is a major factor in maternal deaths and a deterrent to women accessing health services. It has long-lasting effects beyond the walls of the facility, including psychological effects for women, higher risk of dissolution and violence for families ^2^, and the potential impoverishment of households due to high costs of care ^3^. Poor, less educated, younger, and minority women are less likely to receive good quality reproductive health care ^4^. The quality of facilities may therefore be a catalyst for where health inequities are produced and reproduced, further exacerbating intergenerational inequalities in health ^5^.

One aspect of quality that needs to be addressed is the person-centered dimension of quality, the most overt form of which is the mistreatment of women in health facilities. In many parts of the globe, women are hit, slapped, shouted at, and abandoned during childbirth ^6^. The Universal Rights of Childbearing Women Charter denounces such acts through its declaration: “Every woman has a right to dignified, respectful, sexual and reproductive health, including during childbirth” ^7^. Consequently, global movements have called for the need to focus on person-centered care (PCC) ^8^: Engaging women and communities in health care to improve the quality of patient experience and patient-provider interactions.

Adapted from the definition by the Institute of Medicine, we define person-centered reproductive health care (PCRHC) as: “Providing reproductive health care that is respectful of and responsive to individual women and their families’ preferences, needs and values, and ensuring that their values guide all clinical decisions” ^9^. PCRHC needs more emphasis, both as an indicator of human rights and a valued quality domain, and for its association with better health outcomes ^10^. For example, PCC aspects of quality, such as information-sharing and interpersonal relations, are correlated with increased adoption and continuation of modern family planning methods ^11^. Communication between women and their provider during prenatal and delivery care strongly determines women’s satisfaction and utilization of services ^12, 13^. Continuous support during labor and delivery from partners and providers, including companions of choice, is associated with shorter labor, better coping with pain, decreased incidence of operative birth, increased incidence of spontaneous vaginal delivery, increased maternal satisfaction, less anxiety, and increased rates of breastfeeding initiation ^13, 14^. Moreover, important predictors of women’s satisfaction with care during delivery in health facilities have been identified as respect, politeness, friendliness, emotional support by a birth companion, privacy, and cleanliness of facilities ^15^. While global initiatives have begun to address PCC for women’s health ^16^, there is a lack of consensus on how it relates to clinical aspects of quality, how to measure PCC, and how to apply these measures across different contexts.

The objective of this paper is to review theories related to PCRHC, and to develop a framework as it relates to improving the quality of reproductive health, particularly in LMICs. This paper proposes a new framework called the “Person-Centered Care Framework for Reproductive Health Equity” that lays out the dimensions of PCC and the ways in which it links with clinical quality of care in facilities and broader factors at the community and national level.

## Understanding person-centered reproductive health care: definitions and measures

While PCC has received increased attention in developed settings, there is no consensus on how to measure it for reproductive health outcomes. Thus, in order to define PCRHC and identify unifying measures for it, we assessed separate bodies of work that discuss overlapping issues related to PCC, identified from PubMed and other databases as part of a forthcoming systematic review on measures of maternal health person-centered care (unpublished study, Nicholas Rubashkin, Nadia Diamond-Smith, Ruby Warnock; UCSF). This includes literature from health system responsiveness ^17– 19^, perceived quality of care ^20, 21^, mistreatment of women during childbirth ^6, 22, 23^, and the general literature on quality of care for maternal health ^24– 28^ and family planning ^29, 30^. In addition, we examined the general literature on PCC, mostly from developed settings ^9, 31– 33^. These separate bodies of work include important aspects of PCC, yet are framed differently.

The World Health Survey module on health system responsiveness (HSR) takes a broader focus on non-clinical measures of how individuals are treated and the environment in which treatment occurs in health facilities; however, it is not specific for reproductive health. Domains from the HSR include autonomy, dignity, confidentiality of personal information, quality of basic amenities, choice, prompt attention, clarity of communication, and social support ^17– 19^. The literature on mistreatment, on the other hand, tends to be specific to care during childbirth, but is framed in the negative—disrespect and abuse, which is described as treatments that make women feel humiliated or disrespected ^22^. Bohren *et al.* identify the following typologies for mistreatment of women during childbirth: Physical abuse, sexual abuse, verbal abuse, stigma and discrimination, failure to meet professional standards of care, poor rapport between women and providers, health system conditions and constraints, and inappropriate demands for payment ^6^. The literature on mistreatment of women has spurred a lot of work around respectful maternity care ^7^. PCRHC, however, captures, and extends beyond, respectful maternity care.

We also explored previous measures of quality of care and PCC in the family planning literature, identified from PubMed, including (but not limited to) survey tools, such as the Service Provision Assessment, Balanced Counseling Strategy, and Quick Investigation of Quality, and measures used by individual studies ^11, 34– 36^. Based on these literatures, we identified eight domains of PCRHC, described below. These domains when put into practice, would each encompass specific measures (see Table 1).

**Table 1.** Domains and definitions for person-centered care.

**Dignity**	Dignity refers to the ability of women to receive care in a respectful and caring setting. It captures the typologies of physical and verbal abuse from the literature on mistreatment of women during labor and delivery, as well as less subtle acts during patient-provider encounters that make women and their families feel disrespected.
**Autonomy**	Autonomy implies that providers of health services respect women’s views of what is appropriate and support women, her family, and companion of choice to make informed choices. This includes providing consented care. An example of a measure for autonomy is whether women feel involved in decision-making about their care and whether their permission is sought before treatments.
**Privacy/Confidentiality**	This relates to privacy in the environment in which care is provided, and the concept of privileged communication and confidentiality of medical records. An example is whether women feel others who are not involved in their care could hear information about their care or could see them during physical examinations or during labor and delivery without physical examinations.
**Communication**	This domain refers to providers clearly explaining to women and family the nature of their condition, details of treatment, and available treatment options. An example is whether providers clearly explain to women their conditions and the purpose of treatments, any side effects of treatments, and whether women understand explanations.
**Social support**	This domain reflects the extent to which women have access to their companion of choice when receiving care. It also includes their right to receive food and other consumables from family where deemed appropriate. An example is whether family and friends are allowed to stay with them during care.
**Supportive care**	This refers to providers providing care in a timely, compassionate and caring manner, as well as integration of care in a way that is responsive to patient needs. It also captures abandonment or denial of care, protection from harm and unnecessary procedures, and patient safety. It includes women’s perceptions of how providers respond to them when they need more help.
**Trust**	This captures how women assess their care with providers. Here, measures include whether women feel providers tell them the truth about their care, their health, their child, their situation, and whether they have confidence in the competence of their providers.
**Health facility environment**	This captures the quality of the facility and providing a fully enabled environment, including the commodities and equipment, but also referral system, communication and transportation, maternal and neonatal health team that can cover the full continuum of care, environment where staff are respected and valued and that is clean, and the extent to which a health facility offers a welcoming and pleasant environment. Examples include clean surroundings and enough space in waiting rooms and wards.

These domains are not mutually exclusive. For instance, autonomy depends on communication, while trust may depend on perceptions of supportive care as well as communication. Nonetheless, the domains provide a comprehensive map for developing measures that capture key aspects of PCRHC. While certain components of PCC are based in human rights and should be part of universal standards of practice, there are cultural differences in terms of expectations of care. These domains may be used to develop measures that are culturally relevant and uphold basic human rights.

## Grounding person-centered care in existing frameworks and theory

To develop a framework for PCRHC, we started with the general PCC literature, which has been widely applied in nursing care ^37– 39^. In particular, McCormack and McCance’s (2006) framework comprises four embedded constructs: 1) *Prerequisites,* which focuses on the attributes of the provider; 2) *The care environment*, including supportive systems, effective staff relationships, and organizational systems; 3) *Person-centered processes*, including working with patient’s beliefs and values, engagement, having sympathetic presence, sharing decision-making and providing for physical needs. These activities influence the fourth construct, *person-centered outcomes*, such as satisfaction and involvement with care. In this framework, PCC is described both as a process and outcome ^38^. However, this framework does not show how PCC relates to clinical care.

The World Health Organization (WHO)’s Quality of Care framework for maternal and newborn health helps to address this link, as it describes how person-centered outcomes relate to clinical quality. In addition, it illustrates how broader health systems lead to the quality of care in facilities, ultimately impacting individual and facility-level outcomes ^40^. The framework describes quality of care in terms of provision of care and experiences of care, and posits a bidirectional process between provision of care and experiences of care, which ultimately leads to outcomes including person-centered outcomes and key maternal and newborn health outcomes. This framework conceptualizes PCC specifically as an outcome.

Addressing the social and cultural determinants of health is important in eliminating health inequities—the systematic differences in health status of different population groups that are avoidable or unnecessary ^41^. While these frameworks are useful in understanding quality of care, they neglect how women may experience differential treatment based on their social status, influences of communities, and more distal factors, such as gender and violence norms and women’s roles in society. Cultural Health Capital (CHC) theory fills this gap and has been used to explore how social status and communities may affect PCC ^42^. Rooted in Bourdieu’s concepts of cultural capital ^43, 44^, CHC is defined as a “specialized set of cultural skills, behaviors and interactional styles that are valued and leveraged as assets by both patients and providers in clinical encounters” ^45^. CHC is related to the concept of social capital, seen as a resource that patients are able to use to improve interactions in the healthcare setting. Importantly, CHC develops over time and is deeply embedded in patients’ past experiences with healthcare providers or perceptions of healthcare institutions—these are a learned set of skills based on practice and experience. Women in lower social standing groups, including the poor, unmarried, and less educated, may not have past experiences with healthcare settings in order to develop CHC. PCC, therefore, is more challenging in contexts where CHC is low ^42^, further deepening health inequities.

The role of expectations of care is reflected in various frameworks on health-seeking behavior. For example, Thaddeus and Maine’s model on the three delays that contribute to maternal mortality among women with complications ^46^, as well as its expansion by Gabrysch and Campbell to include care-seeking for uncomplicated pregnancies, all include the role of perceptions of quality care as well as sociocultural factors, economic and physical accessibility ^47^. The Disparities in Skilled Birth Attendance framework further expands on previous work by highlighting that disparities in three important determinants: *Perceived need* for care, *perceived accessibility* (physical and financial) of the service, and *perceived quality* of care contribute to disparities in use of skilled birth attendants ^48^. Finally, past frameworks have highlighted the importance of societal/national factors in understanding health equity and maternal healthcare. A framework developed by Freedman and Kruk (2014) for understanding respectful care during childbirth discussed how system level “deficiencies” that are seen as normal and accepted can lead to poor treatment of women ^49^. Jewkes and Penn-Kekana conclude that violence against women in obstetric care settings is the result of broader gender inequality, which places women in subordinate positions and creates normative power differentials between providers and patients ^50^. While most of the current evidence focuses on childbirth, we believe that the same factors apply more broadly to reproductive health issues, including family planning and safe abortion.

## Towards a conceptual framework: the person-centered care framework for reproductive health equity

**Figure 1.**
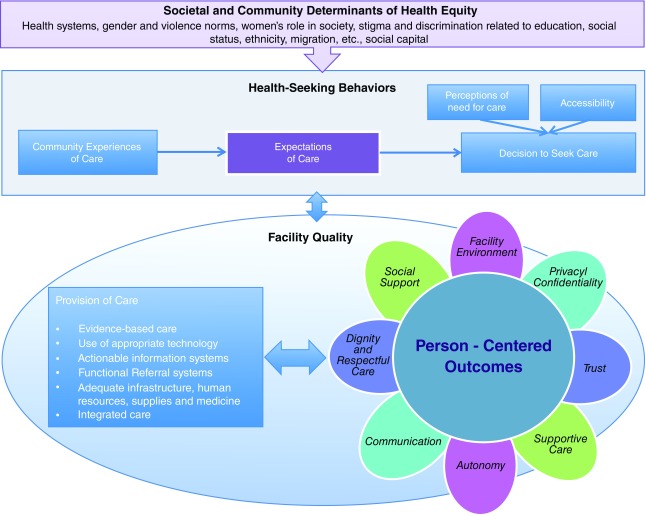
Person-Centered Care Framework for Reproductive Health Equity.

The Person-Centered Care Framework for Reproductive Health Equity builds on these existing frameworks, theories, and literature to situate the domains of PCC ( Figure 1). The Framework has a number of assumptions. First, there are three levels of interacting contexts at play in achieving reproductive health equity. The three levels include: 1) Societal and community determinants of health equity; 2) women’s health-seeking behaviors; and 3) facility-level factors, including the provision of technical care and the person-centered dimensions of care. Second, there is a bidirectional influence between health-seeking behaviors and quality of care women experience in facilities. We assume that not only does the decision to seek care influence women’s experiences in the facility, but that the quality of care in the facility will also influence communities’ and individuals’ perceptions of care, needs for care, expectations of care, and ultimately seeking care. Lastly, building off WHO’s quality of care framework ^40^, the Framework also assumes that there is a bidirectional relationship between provision of care and PCC.

Determinants of health equity include broader health systems, gender and violence norms, women’s role in society, stigma and discrimination related to education, social status, ethnicity, and social capital. The way in which women are treated in healthcare settings and in communities in general is oftentimes a reflection of much broader, societal level norms, policies, and behaviors. In settings where women have low status in the household or community, or there is societal acceptance of differential treatment or discrimination based on socio-economic status, such as racial/ethnic minority groups or social statuses, it is possible that there is more normative acceptance of poor treatment of women in the health facility.

These determinants of health equity influence women’s health-seeking behaviors, including expectations of and decision to seek care. In line with CHC theory, if women have never been to a healthcare facility prior to their delivery, as is common in India, where less than a third of women go to three or more antenatal care visits ^51^, their expectations are likely to differ significantly from a woman who has had past experiences with formal healthcare. Low levels of awareness and realization of deficiencies in services due to illiteracy often leads to lower expectation of the health system ^52^. It is likely that women living in high poverty communities are influenced by their first experience of institutional care, and that their expectations diminish or change with increasing familiarity with the system. Importantly, the experience of care at the facility shapes community’s perception, expectations of care, and ultimately, whether a woman chooses to go to a facility or not ^53^. In developing countries, women with complications delay or avoid seeking care in the same facility if they had a previous negative experience with a provider ^52, 54^. Thus, women may be less likely to: 1) Seek care; 2) have high expectations of care; and 3) have skills and resources to navigate the system and demand better care.

At the facility level, we build on the WHO’s Quality of Care framework by expanding on experiences of care and specifying the domains of PCC. While under-developed in the literature, providers also have constraints in providing quality maternity care, including low salary, lack of recognition, restrictions on clinical practice, lack of supplies and equipment, moral distress and burn out ^55^. In addition, there is a feedback loop from the facility-level to community perceptions. For example, women’s experiences of care, whether positive or negative, will be fed back to their sisters, neighbors, daughters, and friends, thus influencing community perceptions of facilities, expectations of care, and ultimately, whether a woman chooses to go to a facility or not ^53^.

## Conclusion

Given the interconnectedness of women’s health to the broader Sustainable Development Goal (SDG) agenda, new frameworks are needed to achieve health equity in quality of care. For example, of the 17 SDGs, only one is health related (SDG 3); however, many argue that progress against any and all of the SDGs will be delayed without advancements in related goals, including women’s empowerment, poverty, water and sanitation, and quality education ^56– 58^. The framework proposed in this paper integrates mutually interdependent factors at three levels to explain potential sources of health inequity in quality of care, and can also be used to find solutions and next steps. Overarching determinants of health equity influence health-seeking behaviors, such as community’s experiences of care, expectations and decision to seek care, but also further determine the provision of care within the facility. We propose domains of PCC, and situate PCC not only as a result of provision of care (primarily the structural and clinical aspect in facility environment), but also the process of care that can further enhance the service provision.

The Framework can be used to inform future strategies and interventions to improve PCC for women, families, and communities. First, it describes how the facility can be a context in which health inequities can be mitigated, particularly through improving PCC. Specifically, health facilities should deliver care that engages women and family caregivers at all levels of care. There are challenges to engaging certain subgroups of women, including those who are poor and less educated, because of provider-patient power dynamics ^59^, language barriers, and low cultural health capital in the health care setting ^42^. Efforts to address these barriers are essential to mitigating health inequities. The domains in the Framework may be used as guiding principles for facilities or policies aiming to improve PCC, focusing on dignity and respectful care, autonomy, privacy and confidentiality, improving patient-provider communication, social support throughout care, timely and compassionate care, and ensuring that women are treated equally regardless of social status and socioeconomic background. While the Framework is currently based on women’s experiences during childbirth and receiving family planning services, its domains and guiding principals are likely applicable to other components of reproductive and maternal health care, including, but not limited to, preconception, antenatal, abortion, and sexually transmitted infections (STIs) care.

It is important to note that providers in facilities also need support from policy-makers and health systems to provide high quality maternity care. Providers are also reflective of larger social and economic factors, oftentimes themselves disrespected for being single and/or working, experiencing lack of safety and security in communities, and absent from policy dialogue ^55^. All of these factors combined are significant barriers in providing high quality PCC during maternity and reproductive health care ^55^. Reorienting health systems to better respond to women’s, as well as providers’, needs and preferences through service delivery points in the community could improve mortality and health outcomes ^60^.

Future research should test this Framework in multiple settings in order to better understand women’s experiences of care in different contexts. Importantly, further research is also needed to develop culturally appropriate, context-relevant measures that not only address women’s values and backgrounds, but also reflect international guidelines and basic human rights. Additionally, future interventions need to address how gender and economic inequities at the national level may play out in the facility and how health systems may better support not only women, but health workers who are also operating under contexts of social inequities ^59^.

Across the world, women seek dignity and respect for reproductive health care. Women’s experiences during care need to be visualized in a holistic way, where the parameters to assess and improve the quality of services should not only be restricted to within the facility, but also in broader communities. Adopting a PCC framework directly places women’s values, decision-making, and cultural backgrounds in the center of care. The Framework will be of value in the design and strengthening of service improvements that are responsive to women’s needs and experience. Integrating the components of this Framework with quality improvement processes in LMICs, and also for poor and vulnerable women living in or migrating to high-income countries, has the potential to lead to improved access to and utilization of safe and humane reproductive health services for women.

## References

[1] WHO: Maternal mortality. WHO.2015; [cited 2016 Oct 4]. Reference Source

[2] Koblinsky M Chowdhury ME Moran A: Maternal morbidity and disability and their consequences: neglected agenda in maternal health. *J Health Popul Nutr.* 2012;30(2):124–30. 10.3329/jhpn.v30i2.11294 22838155 PMC3397324

[3] Storeng KT Baggaley RF Ganaba R: Paying the price: the cost and consequences of emergency obstetric care in Burkina Faso. *Soc Sci Med.* 2008;66(3):545–57. 10.1016/j.socscimed.2007.10.001 18061325

[4] Say L Raine R: A systematic review of inequalities in the use of maternal health care in developing countries: examining the scale of the problem and the importance of context. *Bull World Health Organ.* 2007;85(10):812–9. 10.2471/BLT.06.035659 18038064 PMC2636485

[5] Marmot M Allen J Bell R: WHO European review of social determinants of health and the health divide. *Lancet.* 2012;380(9846):1011–29. 10.1016/S0140-6736(12)61228-8 22964159

[6] Bohren MA Vogel JP Hunter EC: The Mistreatment of Women during Childbirth in Health Facilities Globally: A Mixed-Methods Systematic Review. *PLoS Med.* 2015;12(6):e1001847. 10.1371/journal.pmed.1001847 26126110 PMC4488322

[7] The White Ribbon Alliance: Respectful Maternity Care.White Ribbon Alliance.2013; [cited 2016 Oct 5]. Reference Source

[8] ten Hoope-Bender P de Bernis L Campbell J: Improvement of maternal and newborn health through midwifery. *Lancet.* 2014;384(9949):1226–35. 10.1016/S0140-6736(14)60930-2 24965818

[9] Institute of Medicine (US) Committee on Quality of Health Care in America: Crossing the Quality Chasm: A New Health System for the 21st Century.2001; [cited 2013 Jul 25]. 25057539

[10] Griffin SJ Kinmonth AL Veltman MW: Effect on health-related outcomes of interventions to alter the interaction between patients and practitioners: a systematic review of trials. *Ann Fam Med.* 2004;2(6):595–608. 10.1370/afm.142 15576546 PMC1466743

[11] RamaRao S Lacuesta M Costello M: The link between quality of care and contraceptive use. *Int Fam Plan Perspect.* 2003;29(2):76–83. 12783771 10.1363/ifpp.29.076.03

[12] Dettrick Z Firth S Jimenez Soto E: Do strategies to improve quality of maternal and child health care in lower and middle income countries lead to improved outcomes? A review of the evidence. *PLoS One.* 2013;8(12):e83070. 10.1371/journal.pone.0083070 24349435 PMC3857295

[13] Hodnett ED Gates S Hofmeyr GJ: Continuous support for women during childbirth. *Cochrane Database Syst Rev.* 2012;10: CD003766. 10.1002/14651858.CD003766.pub4 23076901 PMC4175537

[14] Berghella V Baxter JK Chauhan SP: Evidence-based labor and delivery management. *Am J Obstet Gynecol.* 2008;199(5):445–54. 10.1016/j.ajog.2008.06.093 18984077

[15] Bhattacharyya S Srivastava A Avan BI: Delivery should happen soon and my pain will be reduced: understanding women’s perception of good delivery care in India. *Glob Health Action.* 2013;6(1): 22635. 10.3402/gha.v6i0.22635 24267316 PMC3838967

[16] White Ribbon Alliance: White Ribbon Alliance. White Ribbon.2016 Reference Source

[17] Rice N Robone S Smith PC: The measurement and comparison of health system responsiveness.HEDG, c/o Department of Economics, University of York; [cited 2013 Jun 20]. Report No.: 08/05.2008 Reference Source

[18] Robone S Rice N Smith PC: Health Systems’ Responsiveness and Its Characteristics: A Cross-Country Comparative Analysis. *Health Serv Res.* 2011;46(6pt2):2079–2100. 10.1111/j.1475-6773.2011.01291.x 21762144 PMC3393001

[19] WHO: The Health Systems Responsiveness Analytical Guidelines for Surveys in the MCSS. WHO.2005; [cited 2013 Jun 20]. Reference Source

[20] van Duong D Binns CW Lee AH: Measuring client-perceived quality of maternity services in rural Vietnam. *Int J Qual Health Care.* 2004;16(6):447–52. 10.1093/intqhc/mzh073 15557354

[21] Haddad S Fournier P Potvin L: Measuring lay people’s perceptions of the quality of primary health care services in developing countries. Validation of a 20-item scale. *Int J Qual Health Care.* 1998;10(2):93–104. 10.1093/intqhc/10.2.93 9690882

[22] Abuya T Warren CE Miller N: Exploring the Prevalence of Disrespect and Abuse during Childbirth in Kenya. *PLoS One.* 2015;10(4):e0123606. 10.1371/journal.pone.0123606 25884566 PMC4401776

[23] Bowser D Hill K: Exploring Evidence for Disrespect and Abuse in Facility-Based Childbirth: Report of a Landscape Analysis | Traction Project.2010; [cited 2015 Aug 31]. Reference Source

[24] Donabedian A: The quality of care. How can it be assessed? *JAMA.* 1988;260(12):1743–8. 10.1001/jama.1988.03410120089033 3045356

[25] Hulton LA Matthew Z Stones RW: A framework for the evaluation of quality care in maternity services. University of Southampton;2000; [cited 2013 Aug 1]. Reference Source

[26] Lawrence HC 3rd Copel JA O’Keeffe DF: Quality patient care in labor and delivery: a call to action. *Am J Obstet Gynecol.* 2012;207(3):147–8. 10.1016/j.ajog.2012.07.018 22939715

[27] Srivastava A Avan BI Rajbangshi P: Determinants of women’s satisfaction with maternal health care: a review of literature from developing countries. *BMC Pregnancy Childbirth.* 2015;15(1):97. 10.1186/s12884-015-0525-0 25928085 PMC4417271

[28] Tunçalp Ö Were WM MacLennan C: Quality of care for pregnant women and newborns-the WHO vision. *BJOG.* 2015;122(8):1045–9. 10.1111/1471-0528.13451 25929823 PMC5029576

[29] Bruce J: Fundamental Elements of the Quality of Care: A Simple Framework. *Stud Fam Plann.* 1990;21(2):61–91. 10.2307/1966669 2191476

[30] Dehlendorf C Henderson JT Vittinghoff E: Association of the quality of interpersonal care during family planning counseling with contraceptive use. *Am J Obstet Gynecol.* 2016;215(1):78.e1–9. 10.1016/j.ajog.2016.01.173 26827879

[31] Bechtel C Ness DL: If you build it, will they come? Designing truly patient-centered health care. *Health Aff (Millwood).* 2010;29(5):914–20. 10.1377/hlthaff.2010.0305 20439880

[32] Berwick DM: What 'patient-centered' should mean: confessions of an extremist. *Health Aff (Millwood).* 2009;28(4):w555–65. 10.1377/hlthaff.28.4.w555 19454528

[33] Jenkinson C Coulter A Bruster S: The Picker Patient Experience Questionnaire: development and validation using data from in-patient surveys in five countries. *Int J Qual Health Care.* 2002;14(5):353–8. 10.1093/intqhc/14.5.353 12389801

[34] Tumlinson K Speizer IS Curtis SL: Accuracy of standard measures of family planning service quality: findings from the simulated client method. *Stud Fam Plann.* 2014;45(4):443–70. 10.1111/j.1728-4465.2014.00007.x 25469929 PMC4258897

[35] Kamhawi S Underwood C Murad H: Client-centered counseling improves client satisfaction with family planning visits: evidence from Irbid, Jordan. *Glob Health Sci Pract.* 2013;1(2):180–92. 10.9745/GHSP-D-12-00051 25276531 PMC4168569

[36] Askew I Mensch B Adewuyi A: Indicators for measuring the quality of family planning services in Nigeria. *Stud Fam Plann.* 1994;25(5):268–83. 10.2307/2138058 7871552

[37] Koren MJ: Person-centered care for nursing home residents: the culture-change movement. *Health Aff (Millwood).* 2010;29(2):312–7. 10.1377/hlthaff.2009.0966 20056692

[38] McCormack B McCance TV: Development of a framework for person-centred nursing. *J Adv Nurs.* 2006;56(5):472–9. 10.1111/j.1365-2648.2006.04042.x 17078823

[39] Brownie S Nancarrow S: Effects of person-centered care on residents and staff in aged-care facilities: a systematic review. *Clin Interv Aging.* 2013;8:1–10. 10.2147/CIA.S38589 23319855 PMC3540911

[40] Tunçalp Ö Were WM MacLennan C: Quality of care for pregnant women and newborns-the WHO vision. *BJOG.* 2015;122(8):1045–9. 10.1111/1471-0528.13451 25929823 PMC5029576

[41] Kawachi I Subramanian SV Almeida-Filho N: A glossary for health inequalities. *J Epidemiol Community Health.* 2002;56(9):647–52. 10.1136/jech.56.9.647 12177079 PMC1732240

[42] Dubbin LA Chang JS Shim JK: Cultural health capital and the interactional dynamics of patient-centered care. *Soc Sci Med.* 2013;93:113–120. 10.1016/j.socscimed.2013.06.014 23906128 PMC3887515

[43] Bourdieu P: The Forms of Capital. In: *Handbook of Theory and Research for the Sociology of Education* New York: Greenwood Press;1986;241–58. Reference Source

[44] Bourdieu P: The Logic of Practice. Stanford: Stanford University Press;1980 Reference Source

[45] Shim JK: Cultural health capital: A theoretical approach to understanding health care interactions and the dynamics of unequal treatment. *J Health Soc Behav.* 2010;51(1):1–15. 10.1177/0022146509361185 20420291 PMC10658877

[46] Thaddeus S Maine D: Too far to walk: maternal mortality in context. *Soc Sci Med.* 1994;38(8):1091–110. 10.1016/0277-9536(94)90226-7 8042057

[47] Gabrysch S Campbell OM: Still too far to walk: literature review of the determinants of delivery service use. *BMC Pregnancy Childbirth.* 2009;9(1):34. 10.1186/1471-2393-9-34 19671156 PMC2744662

[48] Afulani PA Moyer C: Explaining Disparities in Use of Skilled Birth Attendants in Developing Countries: A Conceptual Framework. *PLoS One.* 2016;11(4):e0154110. 10.1371/journal.pone.0154110 27105309 PMC4841546

[49] Freedman LP Kruk ME: Disrespect and abuse of women in childbirth: challenging the global quality and accountability agendas. *Lancet.* 2014;384(9948):e42–4. 10.1016/S0140-6736(14)60859-X 24965825

[50] Jewkes R Penn-Kekana L: Mistreatment of Women in Childbirth: Time for Action on This Important Dimension of Violence against Women. *PLoS Med.* 2015;12(6):e1001849. 10.1371/journal.pmed.1001849 26126175 PMC4488340

[51] International Institute for Population Sciences (IIPS) and Macro International: National Family Health Survey (NFHS-3), 2005–06. India: Mumbai: IIPS;2007; **1** Reference Source

[52] Das P Basu M Tikadar T: Client satisfaction on maternal and child health services in rural bengal. *Indian J Community Med.* 2010;35(4):478–81. 10.4103/0970-0218.74344 21278865 PMC3026123

[53] Kyomuhendo GB: Low Use of Rural Maternity Services in Uganda: Impact of Women’s Status, Traditional Beliefs and Limited Resources. *Reprod Health Matters.* 2003;11(21):16–26. 10.1016/S0968-8080(03)02176-1 12800700

[54] Srivastava A Avan BI Rajbangshi P: Determinants of women’s satisfaction with maternal health care: a review of literature from developing countries. *BMC Pregnancy Childbirth.* 2015;15:97. 10.1186/s12884-015-0525-0 25928085 PMC4417271

[55] Filby A McConville F Portela A: What Prevents Quality Midwifery Care? A Systematic Mapping of Barriers in Low and Middle Income Countries from the Provider Perspective. *PLoS One.* 2016;11(5):e0153391. 10.1371/journal.pone.0153391 27135248 PMC4852911

[56] Requejo JH Bhutta ZA: The post-2015 agenda: staying the course in maternal and child survival. *Arch Dis Child.* 2015;100(Suppl 1):S76–81. 10.1136/archdischild-2013-305737 25613979

[57] United Nations: Proposal for Sustainable Development Goals: Sustainable Development Knowledge Platform. [cited 2016 Oct 4]. Reference Source

[58] Norheim OF Jha P Admasu K: Avoiding 40% of the premature deaths in each country, 2010–30: review of national mortality trends to help quantify the UN sustainable development goal for health. *Lancet.* 2015;385(9964):239–52. 10.1016/S0140-6736(14)61591-9 25242039

[59] Andersen HM: “Villagers”: differential treatment in a Ghanaian hospital. *Soc Sci Med.* 2004;59(10):2003–12. 10.1016/j.socscimed.2004.03.005 15351468

[60] World Health Organization: Primary Health Care: Now More Than Ever. WHO.2008, [cited 2016 Sep 26]. Reference Source

