# Supplementary material for: Advancing a conceptual model to improve maternal health quality: The Person-Centered Care Framework for Reproductive Health Equity
Source: Gates Open Res. 2017 Nov 6;1:1. [Version 1] doi: 10.12688/gatesopenres.12756.1 (PMC5764229; doi:10.12688/gatesopenres.12756.1)
Supplement: Peer review history

# Peer review: Referee response for version 1

**DOI:** 10.21956/gatesopenres.13816.r26134

**Published:** 2018-01-10

**Authors:** Charlotte E. Warren

**Affiliations:** 1 Population Council, Washington, DC, USA

Thank you for the opportunity to review this paper. A very interesting concept and very timely given the increased focus on equity and women’s experience and perceptions of quality of care. The framework definitely does require testing. The domains described are challenging to measure. This was one of the biggest challenges in the initial studies measuring the prevalence of disrespect and abuse during childbirth… moving from a description to a definition that can be measured. See Freedman et al. ^1^

 I have a few minor comments that should not hold the paper from being indexed.

- **Introduction third paragraph: **The authors write: “Communication between women and their provider during prenatal and delivery care strongly determines women’s satisfaction and utilization of services” - What about satisfaction and use of PNC? Although not so common women do access PNC.
- **Introduction** **7 ^th^ paragraph** – the Balanced Counseling Strategy is not a survey tool it is a counseling tool for FP providers. 
- **Table 1:** Domains and definitions for person-centered care. It would be helpful to use the same language /format for each domain - i.e. “examples include….”. Most of the domains have explicit examples of how they are measured – but for three (dignity, trust and supportive care) are not so clear.

I have read this submission. I believe that I have an appropriate level of expertise to confirm that it is of an acceptable scientific standard.

## References

[1] FreedmanLPRamseyKAbuyaTBellowsBNdwigaCWarrenCEKujawskiSMoyoWKrukMEMbarukuG: Defining disrespect and abuse of women in childbirth: a research, policy and rights agenda. *Bull World Health Organ*.2014;92(12) : 10.2471/BLT.14.137869 915-7 10.2471/BLT.14.137869 25552776PMC4264393

# Peer review: Referee response for version 1

**DOI:** 10.21956/gatesopenres.13816.r26135

**Published:** 2018-01-10

**Authors:** Kelsey Holt

**Affiliations:** 1 Department of Family and Community Medicine, University of California, San Francisco, CA, USA; 2 Department of Global Health and Population, Harvard T.H. Chan School of Public Health, Boston, MA, USA

Many thanks for the opportunity to review this exciting commentary. I have detailed below a number of revisions that I feel would strengthen the paper. I am happy to discuss further with the authors if they are interested.

1. Introduction:
  1. The article rightfully makes the case for a framework that focuses on reproductive health broadly, not just maternal health, while acknowledging that the framework is based primarily on the MH and FP literature due to the dearth of PCC concepts/frameworks originating in other areas of RH. However, the introduction (and title) still primarily focuses on maternal health. I recommend broadening the intro and justification for the piece by engaging with other areas beyond obstetric care – e.g., a complementary case to the examples from maternal health could be made that person-centered care is particularly important in family planning care given a history of coercive policies and practices that have led to distrust among many communities.
  2. Human rights are alluded to in multiple places throughout the article, but a more explicit justification in the Intro section for a focus on PCC from a rights-based standpoint, and not just because of a link to health outcomes or other downstream effects—would strengthen the case.
  3. The sentence in the final paragraph of the Intro that states the objective of the framework is a critical sentence and could be more clearly stated; as it stands, “ to develop a framework as it [PCC] relates to improving the quality of RH” is too vague and also doesn’t convey the unique focus on equity
2. Framework:
  1. The unique contribution of this framework lies in layering the concept of social/economic drivers of health inequities onto person-centered care concepts. However, despite the focus on equity, the framework itself does not depict the processes described in the text whereby these social/economic factors influence patient experience of care. I recommend more “real estate” in the framework be given to depicting these processes (which are described in the text but not clear in the framework as a standalone reference). This could be done first by fleshing out the influence of the social/economic components not only on health seeking behavior but on the way individuals experience care (e.g., if a person experiences ethnic or class-based discrimination, that would affect not only their likelihood of seeking care—which is already well depicted—but also what type of care they receive when they get there, independent of their health seeking behaviors. Right?). The paragraph on CHC in the article is very strong and it would be great to translate the CHC constructs discussed into the actual framework. Second, it seems that the main outcome of interest that I think the framework is trying to convey, unjust differences in experiences of reproductive healthcare, should be explicitly represented in the framework. These modifications would make the framework an extremely important contribution to the literature.
  2. Optionally, two excellent points made in the text could be worked into the framework as well: 1) intergenerational nature of health inequities and 2) the fact that provider constraints in provision of PCC are also impacted by the social/economic determinants (e.g., underpaid/lower class providers are overworked and this influences the care they provide), with a possible citation to the Lancet Commission on Women and Health which also makes this point.
3. Table 1
  1. Table 1 is a helpful addition to the literature and could be further strengthened by adding citations to connect each construct with the literature authors reviewed and summarized in the text of the paper
  2. Along the lines of making the paper less MH-specific, a nod to the seminal Bruce quality framework from FP could be added at some point in the Table (perhaps in the communication construct) to diversify the examples
  3. Trust construct: “assess their care with providers” feels somewhat too general – consider rewording to be more specific to trust
4. Other comments/suggestions:
  1. On page 4, a clearer transition could be made at the beginning of the “Grounding person-centered care in existing frameworks and theory” section to link it with the prior and make clear how the resources described in this section differ from the ones reviewed in the prior section
  2. The first paragraph of the Conclusion section could use work to make a stronger case for the importance of the framework. The connection with the SDGs is unclear, and the final sentence describing the framework is also unclear to me.
5. Additional resources that could be engaged with/cited in the paper:
  1. Dennis, A., Blanchard, K., & Bessenaar, T. (2017) ^1^
  2. Harris S, et al. (2016) ^2^

I have read this submission. I believe that I have an appropriate level of expertise to confirm that it is of an acceptable scientific standard, however I have significant reservations, as outlined above.

## References

[1] DennisABlanchardKBessenaarT: Identifying indicators for quality abortion care: a systematic literature review. *J Fam Plann Reprod Health Care*.2017;43(1) : 10.1136/jfprhc-2015-101427 7-15 10.1136/jfprhc-2015-101427 27172880

[2] ReichenbachLHardeeKHarrisS: Measuring and monitoring quality of care in family planning: are we ignoring negative experiences?. *Open Access Journal of Contraception*.2016; 10.2147/OAJC.S101281 10.2147/OAJC.S101281 PMC568316329386941

# Peer review: Referee response for version 1

**DOI:** 10.21956/gatesopenres.13816.r26040

**Published:** 2017-12-14

**Authors:** Sarika Chaturvedi

**Affiliations:** 1 Centre for Complementary and Integrative Health, Interdisciplinary School of Health Sciences, Savitribai Phule Pune University, Pune, India

This is an interesting and relevant work.

 The proposed framework is comprehensive and appropriately builds on existing literature.

 I have the following comments on the manuscript:

- Although the authors present several determinants of equity, I find that the framework misses affordability aspect. Afford ability of care strongly impacts decisions to seek care and also the type or level of care sought. It would be more appropriate to include this aspect more explicitly in the proposed framework.
- Accountability is another important determinant that does not appear in the proposed framework. Accountability to users can influence experience of care as well as provision of care, including functional quality of care. In my view, a framework for equity would be incomplete without alluding to accountability to users.
- The authors switch between quality of reproductive care and maternity care. It would be useful to be specific and consistent.
- It is not clear what the authors mean by 'integrated care'. It would be useful to clarify this.

I have read this submission. I believe that I have an appropriate level of expertise to confirm that it is of an acceptable scientific standard, however I have significant reservations, as outlined above.

# Peer review: Referee response for version 1

**DOI:** 10.21956/gatesopenres.13816.r26042

**Published:** 2017-11-27

**Authors:** Petra ten Hoope-Bender

**Affiliations:** 1 Technical Adviser Sexual and Reproductive Health and Rights, United Nations Population Fund (UNFPA), Geneva, Switzerland

Very interesting and timely article. Would suggest a few clarifications and inclusion of some aspects of  compassion and empowerment, just to open space for a fuller discussion in a next paper. The touch upon Cultural Health Capital is both interesting and promising for a deeper delve in a possible future article. The PCC Framework makes sense, but needs to be tried and tested to show it really addresses and captures all the important aspects of PCC. In addition, a health system 'return on investment' case study for PCC would make a big difference in helping countries use the Framework as a guiding tool for service improvement or development.

 Please click here to download a PDF of the article with my detailed comments.

I have read this submission. I believe that I have an appropriate level of expertise to confirm that it is of an acceptable scientific standard.
